# Relationship Between Body Roundness Index and Diabetic Kidney Disease in Patients With Type 2 Diabetes Mellitus: A Population-Based Study

**DOI:** 10.1155/jdr/1854458

**Published:** 2025-09-03

**Authors:** Mengdie Chen, Yiyun Wang, Ping Feng, Qidong Zheng, Qiao Liu, Mimi Chen, Chaoyin Lu, Lijing Wu

**Affiliations:** ^1^Department of Endocrinology, Taizhou Central Hospital (Taizhou University Hospital), Zhejiang, China; ^2^Department of Internal Medicine, Yuhuan Second People's Hospital, Zhejiang, China

**Keywords:** biomarker, body roundness index, diabetic kidney disease, obesity, Type 2 diabetes

## Abstract

**Background:** Considering the reported link between obesity and diabetic kidney disease (DKD), this study investigated the association between body roundness index (BRI) and DKD.

**Methods:** Cross-sectional data were obtained from the National Metabolic Management Center (MMC) of Yuhuan Second People's Hospital and Taizhou Central Hospital (Taizhou University Hospital) between September 2017 and May 2024. BRI was calculated using waist circumference (WC) and height. BRI was analyzed as a continuous and categorical variable to examine its association with DKD. Multivariate logistic regression and restricted cubic spline (RCS) analyses of the interplay between the BRI and DKD were conducted. Subgroup analysis was also performed.

**Results:** Among 12,231 analyzed individuals, 5020 (41.0%) exhibited DKD. The BRI of individuals with DKD was higher than that of those without (*p* < 0.001). Compared with those in the first BRI tertile (T1), those in T2 or T3 showed higher DKD prevalence rates (T1 33.4% vs. T2 41.3% vs. T3 48.3%, *p* < 0.001). Compared with individuals in T1, those in T2 and T3 exhibited fully adjusted odds ratios for the relationship between BRI and DKD of 1.25 (95% confidence interval [CI]: 1.13–1.37, *p* < 0.001) and 1.42 (95% CI: 1.28–1.57, *p* < 0.001), respectively. A significant trend across BRI tertiles for DKD odds was observed (*p* for trend < 0.001). RCS regression with three knots at clinically relevant percentiles (5th, 50th, and 95th) confirmed a dose–response relationship between BRI and DKD, demonstrating positive linearity (*p* for nonlinearity = 0.628).

**Conclusion:** BRI demonstrated significant positive associations with DKD, supporting its utility as a clinical risk indicator for early identification of high-risk individuals. Prospective cohort studies are warranted to evaluate BRI's predictive capacity for incident DKD.

## 1. Introduction

Diabetic kidney disease (DKD) is associated with a reduced estimated glomerular filtration rate (eGFR) or evidence of albuminuria [1]. An estimated 8.2% of adults have Type 2 diabetes mellitus (T2DM) [2], among whom 20%–40% are diagnosed with DKD [3]. DKD frequently leads to end-stage renal disease (ESRD), which results in increased mortality in patients with diabetes [4]. ESRD necessitates hemodialysis treatment, which is expensive and imposes heavy social and personal burdens on patients [5]. Efforts to identify the pathogenic factors that lead to DKD are vital for prevention, highlighting the need for clinical efforts to identify and manage DKD-related risk factors.

Obesity is independently associated with the risk of developing DKD. Although body mass index (BMI) is the most frequently utilized metric and its link with DKD risk has been studied [6–8], more recent research has suggested that DKD risk is more closely tied to abdominal obesity than to general obesity [9]. Moreover, BMI is poorly suited to differentiating between lean mass and fat, and it is also unable to evaluate fat distribution [10, 11].

Given the limitations associated with BMI utilization, the relationship between DKD risk and other indices, including waist circumference (WC), waist–hip ratio (WHR), and visceral fat area (VFA), has also been analyzed [12–14]. However, the limitations of WC and WHR include their correlation with BMI and sensitivities to fat distribution, fat percentage, and body size [15]. Although visceral fat can be evaluated using CT or MRI, these procedures are lengthy and costly and, thus, unsuitable for routine use. Therefore, effective indices are needed to assess and gauge DKD risk.

In this context, the body roundness index (BRI) was first proposed in 2013 [16]. As BRI can reflect height (H)-related body roundness as an indicator of abdominal obesity, it is more suitable for assessing body fat distribution. The BRI is closely associated with many diseases, including metabolic syndrome [17], diabetes [18], cardiovascular disease [19], and kidney disease [20].

However, few studies have assessed the links between the BRI and DKD, and none have considered individuals with T2DM. Therefore, in the present study, we investigated the association between BRI and DKD in patients with T2DM.

## 2. Materials and Methods

### 2.1. Study Design and Participants

This cross-sectional investigation analyzed data from the National Metabolic Management Center (MMC) between 2017 and May 2024. The full details of the MMC program have been described previously [21, 22]. The Clinical Research Ethics Committee of Yuhuan Second People's Hospital and Taizhou Central Hospital (Taizhou University Hospital) approved the protocol, all participants provided written informed consent, and this study adhered to the principles of the Declaration of Helsinki.

**Figure 1:**
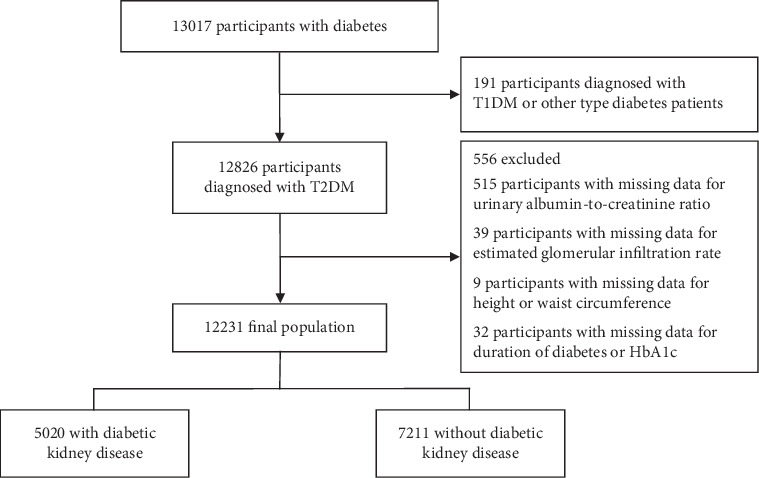
Study flowchart.

This study initially screened 13,017 individuals with diabetes, excluding those with Type 1 diabetes mellitus (T1DM) or other diabetes types (*n* = 191), and those missing eGFR data (*n* = 39), H or WC data (*n* = 9), urinary albumin-to-creatinine ratio (UACR) values (*n* = 515), and details on glycated hemoglobin (HbA1c) levels or diabetes duration (*n* = 32). The final cross-sectional analysis included 12,231 participants. Further details on patient inclusion and exclusion are presented in Figure 1.

### 2.2. Data Collection and Analysis

Trained interviewers utilized a standardized procedure to collect all data from the local MMCs [21], including data related to demographics, lifestyle factors, and medical records. For this study, the analyzed data included patient age, sex, educational level, smoking, alcohol consumption, diabetes duration, diabetes family history, hypertension, dyslipidemia, H, weight (W), WC, hip circumference (HC), VFA, diastolic and systolic blood pressures (DBP and SBP, respectively), and levels of fasting serum C peptide (FCp), fasting blood glucose (FBG), HbA1c, total cholesterol (TC), triglyceride (TG), high-density lipoprotein cholesterol (HDL-C), low-density lipoprotein cholesterol (LDL-C), urea nitrogen (UN), serum creatinine (Scr), uric acid (UA), UACR, and eGFR. A standardized protocol was used to measure H, W, WC, and HC. BMI was calculated as W/H2 (kilograms per square meter), and WHR was calculated as WC/HC. VFA was determined at the umbilical level using a dual bioelectrical impedance analyzer (HDS2000, Omron Healthcare Co.). The BRI was calculated as follows: 364.2–365.5∗√(1 − [WC in centimeters/2*π*]^2^/[0.5∗H in centimeters]^2^), as reported previously [16].

### 2.3. Study Variables

Patients with an SBP ≥ 140 mmHg or a DBP ≥ 90 mmHg were categorized as having hypertension [23], or a previous doctor diagnosis. Similarly, hyperlipidemia was defined as a previous physician diagnosis, or the presence of one or more of the following: TC ≥ 5.7 mmol/L, TG ≥ 1.7 mmol/L, LDL‐C ≥3.6 mmol/L, and HDL‐C < 1.29 mmol/L (females) or < 1.03 mmol/L (males) [24]. Smoking frequencies of almost daily/daily were classified as “yes.” Similarly, alcohol consumption was defined as “yes” in cases of almost weekly/weekly alcohol consumption. Education was assessed as less than or above high school level. Homeostatic model assessment for insulin resistance (HOMA-IR) values were computed as 1.5 + (FCp [pmol/L] × FBG [mmol/L])/2800 [25]. eGFR was determined as previously described [26]. DKD was diagnosed among patients with T2DM with UACR > 30 mg/g and/or eGFR < 60 mL/min/1.73 m^2^ [27].

### 2.4. Statistical Analyses

Categorical variables are presented as proportions (percent), with group comparisons performed using chi-square tests. Normally distributed continuous variables are expressed as means with standard deviations (SD) and compared via one-way analysis of variance (ANOVA). Nonnormally distributed continuous variables are reported as medians with interquartile ranges (IQRs), analyzed using the Kruskal–Wallis tests. Odds ratios (ORs) and 95% confidence intervals (CIs) for the association between BRI and DKD were calculated using logistic regression models. BRI tertiles were treated as ordinal variables to assess trends across tertiles. Model 1 was adjusted for sex and age. Model 2 was additionally adjusted for education, diabetes duration, family history of diabetes, and alcohol consumption/smoking. Finally, Model 3 included HOMA-IR, HbA1c, hypertension history, and dyslipidemia history. Linearity analyses were implemented through a restricted cubic spline (RCS) regression analysis with knots at the 5th, 35th, 65th, and 95th BRI percentiles, allowing the assessment of dose–response relationships between BRI and DKD when using Model 3 adjustment parameters. Stratified analyses were additionally employed to investigate the effects of particular variables on the interplay between BRI and DKD, stratified according to patient sex, age (≤ 55 vs. > 55 years), HbA1c (≤ 7% vs. > 7%), BMI (≤ 25 vs. > 25 kg/m^2^), diabetes duration (≤ 5 vs. > 5 years), and family history of diabetes. Multivariate logistic regression analyses of heterogeneity among subgroups were performed, and likelihood ratio testing was used to examine the interactions between the subgroups and BRI. R 4.3.0 and Free Statistics 1.9.2 were used to perform all analyses. All participants underwent descriptive analyses, in which a two-tailed *p* < 0.05 was considered statistically significant.

## 3. Results

### 3.1. Participant Features

This study enrolled 12,231 individuals (7396 men and 4835 women) who were separated into two groups based on their DKD status, in which 41.0% of the patients were categorized as having DKD. The clinicopathological features of the patients are presented in Table 1. Participants with DKD were often older, had less education, a longer diabetes duration, higher hypertension prevalence, lower eGFR values, no alcohol consumption or family history of diabetes, and had increased DBP, SBP, BMI, WC, BRI, VFA, FBG, FCp, HOMA-IR, HbA1c, UN, Scr, UA, TG, TC, and UACR values compared with individuals without DKD (*p* < 0.001). When the participants were stratified according to BRI tertiles, DKD prevalence gradually trended upward with progressive increases in BRI levels (33.4% vs. 41.3% vs. 48.3%, *p* < 0.001) (Table S1).

### 3.2. Relationship Between BRI and DKD

**Figure 2:**
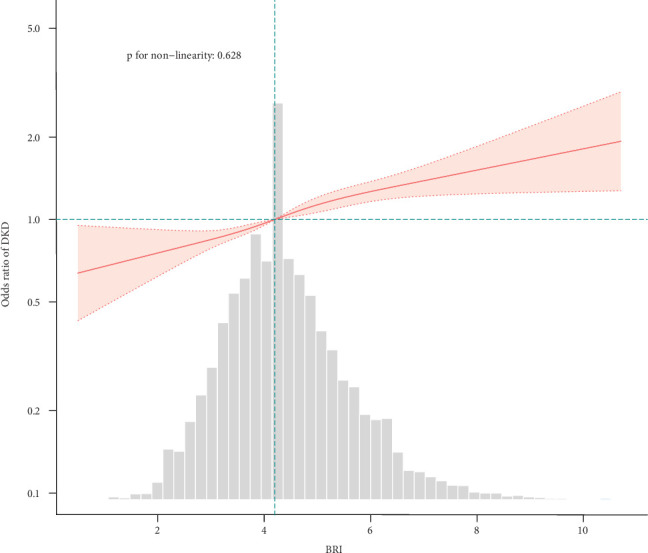
Smooth curve fitting of the BRI-DKD association.

The relationship between BRI and DKD as continuous and categorical variables is presented in Table 2. When BRI was analyzed as a continuous variable, the unadjusted model demonstrated a positive association with greater DKD prevalence (OR = 1.22, 95% CI: 1.19–1.26, *p* < 0.001). It remained statistically significant in all multivariate logistic regression models after adjusting for several covariates including age, sex, diabetes duration, diabetes family history, smoking, alcohol consumption, HOMA-IR, HbA1c, dyslipidemia history, and hyperlipidemia history (Model 1, OR 1.21 [95% CI: 1.17–1.24]; Model 2, OR 1.21 [95% CI: 18–1.25]; and Model 3, OR 1.11 [95% CI: 1.08–1.15]). When tertiles were used to assess the BRI, this index remained significantly positively associated with DKD after adjusting for potential confounding effects. In the unadjusted model, individuals in the last BRI tertile exhibited greater DKD prevalence compared with those in the first tertile (OR = 1.86, 95% CI: 1.7–2.04, *p* < 0.001, Table 2). In Model 3, compared with individuals with BRI T1 (≤ 3.7), those in T2 (> 3.7 and ≤ 4.7) and T3 (> 4.7) exhibited respective ORs of 1.25 (95% CI: 1.13–1.37, *p* < 0.001) and 1.42 (95% CI: 1.28–1.57, *p* < 0.001) (Table 2). A significant trend in DKD prevalence was observed across the BRI tertiles in all models, with RCS analyses revealing a positive linear correlation between the two (*p* for nonlinearity = 0.628) (Figure 2).

### 3.3. Stratified Analyses

**Figure 3:**
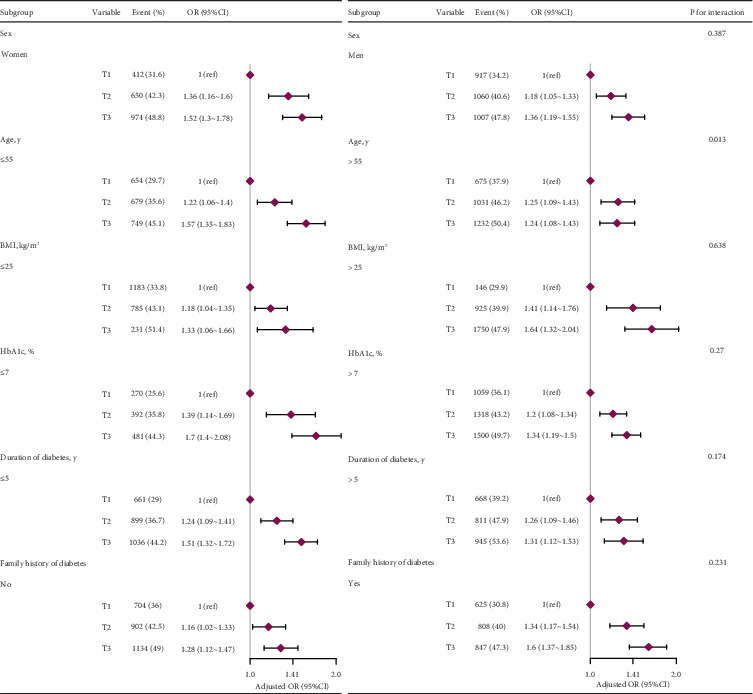
Stratified analyses. Adjusted for age, sex, education, duration of diabetes, diabetes family history, HbA1c, HOMA-IR, hypertension, dyslipidemia, and lifestyle factors (alcohol consumption and smoking) unless those were the variables used for stratification. Abbreviations: OR, odds ratio; CI, confidence interval.

Stratified analyses to examine the impact of specific variables on the interplay between the BRI and DKD revealed no significant interactions when the patients were categorized based on age, sex, BMI, HbA1c level, diabetes duration, or family history of diabetes (Figure 3). Considering multiple testing, *p* < 0.05 for the age interaction might not be statistically significant. In all models, a higher BRI was consistently associated with increased odds of DKD.

## 4. Discussion

In this large population-based cross-sectional study, we clearly exhibited a significant positive relationship between the BRI and DKD, even after controlling for possible confounding effects. Obesity rates have markedly increased in recent decades and have become a worldwide health issue [28]. Obesity is commonly measured using variables such as WC, BMI, and WHR. Although BMI is the most commonly used measure in this context, its inability to evaluate body fat distribution limits its utility. In contrast, WC and WHR cannot distinguish between visceral fat and the more harmful subcutaneous fat. MRI and CT are effective for evaluating visceral fat distribution; however, these procedures are costly, time-consuming, and poorly suited for daily use. These limitations have fueled the development of novel anthropometric indices such as the BRI, which captures H-related body roundness. The effectiveness of the BRI has been demonstrated in studies on metabolic syndrome [17], diabetes [18], cardiovascular disease [19], and kidney disease [20]. Thus, as it reflects the levels of both body and visceral fat, the BRI is an emerging index for health screening and disease risk prediction.

Previous epidemiological studies reported the association between obesity and increased risk of DKD [6–8]. However, most of these studies used BMI for their assessments. For instance, a retrospective longitudinal analysis observed that BMI < 25 kg/m^2^ was closely related to DKD prevention among individuals with T2DM [8]. Additionally, a Mendelian randomization study reported a causal relationship between a higher BMI, increased risk of DKD, and reduced eGFR [6]. Finally, a meta-analysis demonstrated that each 5 kg/m^2^ rise in BMI led to a 16% greater likelihood of developing DKD [7]. However, these results are not universally applicable. One prospective analysis, for instance, reported that a BMI ≥ 25 kg/m^2^ was linked with protection against deteriorating renal function among individuals with T2DM and Stages 3/4 chronic kidney disease [28], although the relatively small size of the sample derived from only one center may have led to bias. Increasing research interest has focused on abdominal obesity–related harm. Consistent with the present results, most reports have demonstrated correlations between abdominal obesity and DKD. Rossi et al. [14] reported an association between WC-based abdominal obesity and a greater risk of microalbuminuria, whereas in other studies, WHR [12] or waist-to-height ratio (WHtR) [29, 30] were more closely linked to DKD risk than the risk attributable to general obesity. Lin et al. noted that VFA increased with DKD progression and was positively associated with prognosis [31]. Additionally, Wu et al. observed a strong relationship between visceral adiposity index (VAI) and DKD [32]. Our previous investigation demonstrated that elevated VAI was independently associated with an increased risk of DKD in elderly patients with T2DM [33]. A separate meta-analysis of 2205 individuals from three cross-sectional studies with available VFA data reported that measures of abdominal obesity, including WC and continuous VFA, were associated with a greater risk of DKD, with patients with T2DM and DKD exhibiting greater odds of being classified as having abdominal obesity, as determined by WC or WHR/WHtR [13]. Man et al. [34], however, found the opposite, observing no association between abdominal obesity and DKD among patients with T2DM. Thus, further studies are required to investigate the interplay between abdominal obesity and DKD. The BRI, a novel anthropometric measure of obesity, has recently emerged as a potential indicator of DKD. Fei et al. [35] and Zhang and Yu [36], utilizing the NHANES database, demonstrated associations between elevated BRI and increased CKD risk among US adults with diabetes and the general US population, respectively. Separately, Ou et al. [37] reported a significant correlation between BRI and albuminuria in a cohort of 1872 Taiwanese T2DM patients. The present study extends this evidence base by examining 12,231 Chinese adults with T2DM—a substantial sample encompassing diverse clinical characteristics and metabolic parameters. This design provides new perspectives on BRI's applicability across ethnic and geographical contexts. To comprehensively evaluate the BRI-DKD relationship, we employed multivariable logistic regression alongside RCS analysis. Further robustness was established through extensive subgroup analyses. Collectively, this methodological approach offers a multifaceted examination of BRI's clinical relevance to DKD.

Although the pathophysiology of BRI in DKD requires further study, several known mechanisms may explain the effects observed in the present study. Adiposity, especially in the abdomen, can contribute to the loss of body homeostasis through the secretion of lipids and inflammatory mediators, ultimately resulting in the progression of the pathological processes that underlie DKD [38, 39]. Obesity is often concurrent with both glomerular and mesangial kidney lipid deposition. Glomerular adiposity can trigger the upregulation of SREBP proteins, leading to mesangial cell proliferation, podocyte apoptosis, and cytokine expression [40, 41]. Obesity also coincides with the accumulation of ectopic fat in the pararenal renal or vascular sinuses, which may further contribute to DKD development [42]. Adipokines, including interleukin-6 (IL-6), tumor necrosis factor-alpha (TNF-*α*), and free fatty acids, can lead to insulin resistance, oxidative stress, and mitochondrial dysfunction that ultimately lead to renal damage [43]. As a proinflammatory cytokine with multiple functions, TNF-*α* is involved in inflammatory activity and DKD-associated insulin resistance [44], whereas IL-6 and leptin can directly affect renal and endothelial function [45]. The correlation between obesity and chronic low-grade inflammation may be associated with renal damage [46]. Abdominal obesity is linked to altered renal hemodynamics and sodium retention, including elevated eGFR and intraglomerular pressure [47, 48]. Overly high levels of visceral adiposity can result in inappropriate renin–angiotensin–aldosterone system activity, contributing to interstitial fibrosis and endothelial activity dysfunction [49, 50], leading to DKD progression. Obesity can induce insulin resistance [51], leading to greater sodium reabsorption and glomerular hyperfiltration, which contribute to severe renal damage [52]. Abdominal obesity is also frequently linked to other types of metabolic dysfunctions that contribute to DKD development [53, 54].

However, this study has several limitations. First, because these results were based on a survey of Chinese adults, their generalizability remains unknown. Moreover, despite efforts to control for several covariates and implement regression models and stratified analyses, residual confounding is possible owing to the effects of variables not considered. In addition, only the correlation between the BRI and overall DKD was assessed, without any corresponding investigation of its relationship with DKD progression, treatment, or prognostic outcomes. Future in-depth studies exploring these relationships and other follow-up outcomes are required. Finally, the cross-sectional design prevented the assessment of a causal link between the BRI and DKD, thus providing another important focus for future prospective studies.

## 5. Conclusions

Overall, the results of this study revealed a strong relationship between BRI and DKD, in which the prevalence of DKD was greater among Chinese patients with T2DM with a higher BRI. Thus, implementing appropriate monitoring programs for individuals with overweight or obesity may help prevent DKD and aid in early diagnosis, thereby reducing the burdens associated with these conditions. These results also serve as a foundation for future randomized controlled trials to explore the effects of reducing the BRI on concomitantly decreasing the incidence of DKD and slowing its progression.

## Figures and Tables

**Table 1:** Clinical features of participants.

**Variables**	**Total (** **n** = 12, 231**)**	**Without DKD (** **n** = 7211**)**	**With DKD (** **n** = 5020**)**	**p** ** value**
Sex, *n* (%)				0.053
Female	4835 (39.5)	2799 (38.8)	2036 (40.6)	
Male	7396 (60.5)	4412 (61.2)	2984 (59.4)	
Age (years), medians (IQR)	55.0 (47.0, 63.0)	54.0 (46.5, 62.0)	57.0 (49.0, 66.0)	< 0.001
Education, *n* (%)				< 0.001
Below high school	10,193 (83.4)	5818 (80.7)	4375 (87.2)	
High school education and above	2035 (16.6)	1390 (19.3)	645 (12.8)	
DBP (mmHg), medians (IQR)	75.0 (69.0, 83.0)	75.0 (69.0, 82.0)	76.0 (70.0, 85.0)	< 0.001
SBP (mmHg), medians (IQR)	130.0 (120.0, 141.0)	127.0 (118.0, 138.0)	134.0 (123.0, 147.0)	< 0.001
BMI (kg/m^2^), medians (IQR)	25.2 (23.1, 27.6)	25.0 (22.9, 27.3)	25.4 (23.2, 27.9)	< 0.001
WC (cm), medians (IQR)	89.2 (83.0, 96.0)	88.6 (82.6, 95.0)	90.0 (84.1, 97.0)	< 0.001
HC (cm), medians (IQR)	95.0 (90.4, 100.0)	95.0 (90.3, 99.0)	95.0 (91.0, 100.0)	< 0.001
WHR, medians (IQR)	0.9 (0.9, 1.0)	0.9 (0.9, 1.0)	0.9 (0.9, 1.0)	< 0.001
BRI, medians (IQR)	4.2 (3.5, 5.1)	4.1 (3.4, 4.9)	4.4 (3.7, 5.3)	< 0.001
VFA (cm^2^), medians (IQR)	94.0 (65.9, 123.7)	90.0 (62.0, 118.0)	100.8 (71.0, 131.0)	< 0.001
Duration of diabetes (years), medians (IQR)	3.4 (0.2, 10.2)	2.8 (0.1, 8.6)	4.6 (0.6, 11.2)	< 0.001
History of hypertension, *n* (%)	5517 (45.1)	2748 (38.1)	2769 (55.2)	< 0.001
History of dyslipidemia, *n* (%)	3325 (27.2)	1944 (27)	1381 (27.5)	0.506
Family history of diabetes, *n* (%)	5840 (47.7)	3560 (49.4)	2280 (45.4)	< 0.001
Smoking, *n* (%)	3591 (29.5)	2119 (29.5)	1472 (29.5)	0.966
Alcohol consumption, *n* (%)	4284 (35.2)	2630 (36.7)	1654 (33.1)	< 0.001
FBG (mmol/L), medians (IQR)	8.2 (6.6, 11.1)	7.9 (6.5, 10.3)	8.8 (6.8, 12.3)	< 0.001
FCp (ng/mL), medians (IQR)	2.1 (1.5, 2.9)	2.0 (1.4, 2.7)	2.3 (1.6, 3.1)	< 0.001
HOMA-IR, medians (IQR)	3.6 (2.9, 4.7)	3.5 (2.8, 4.3)	4.0 (3.1, 5.3)	< 0.001
HbA1c (%), medians (IQR)	8.2 (6.9, 10.2)	8.0 (6.8, 9.9)	8.6 (7.1, 10.5)	< 0.001
UN (mmol/L), medians (IQR)	5.3 (4.4, 6.5)	5.1 (4.3, 6.1)	5.6 (4.5, 7.1)	< 0.001
Scr (mmol/L), medians (IQR)	63.0 (52.0, 76.0)	62.0 (52.0, 72.0)	66.0 (52.0, 84.0)	< 0.001
eGFR (mL/min/1.73 m^2^), medians (IQR)	104.4 (85.4, 125.5)	106.8 (91.0, 125.7)	99.5 (73.4, 125.2)	< 0.001
UA (mmol/L), medians (IQR)	328.0 (268.0, 397.0)	322.0 (266.0, 383.0)	340.0 (273.0, 419.0)	< 0.001
TG (mmol/L), medians (IQR)	1.6 (1.1, 2.4)	1.5 (1.0, 2.2)	1.7 (1.1, 2.5)	< 0.001
TC (mmol/L), medians (IQR)	5.1 (4.3, 5.9)	5.0 (4.3, 5.8)	5.2 (4.3, 6.0)	< 0.001
HDL-C (mmol/L), medians (IQR)	1.1 (0.9, 1.3)	1.1 (0.9, 1.3)	1.1 (0.9, 1.3)	< 0.001
LDL-C (mmol/L), medians (IQR)	2.9 (2.2, 3.5)	2.9 (2.2, 3.5)	2.9 (2.2, 3.6)	0.744
UACR (mg/g), medians (IQR)	20.1 (8.9, 58.7)	10.6 (6.1, 17.3)	77.6 (43.4, 202.4)	< 0.001

*Note:* Data are counts (percent) or medians (IQR) and were, respectively, compared with chi-square and Mann–Whitney *U* tests when comparing the baseline features of those individuals with and without DKD.

Abbreviations: BMI, body mass index; DBP, diastolic blood pressure; DKD, diabetic kidney disease; eGFR, estimated glomerular filtration rate; FBG, fasting blood glucose; FCp, fasting serum C peptide; HbA1c, glycated hemoglobin; HDL-C, high-density lipoprotein cholesterol; HOMA-IR, homeostasis model assessment of insulin resistance; LDL-C, low-density lipoprotein cholesterol; SBP, systolic blood pressure; Scr, serum creatinine; TC, total cholesterol; TGs, triglycerides; UA, uric acid; UACR, urinary albumin-to-creatinine ratio; UN, urea nitrogen; VFA, visceral fat area; WC, waist circumference; WHR, waist-to-hip ratio.

**Table 2:** The relationship between BRI and DKD.

**DKD**	**OR (95% CI)**								
	**No.**	**Crude**	**p** ** value**	**Model 1**	**p** ** value**	**Model 2**	**p** ** value**	**Model 3**	**p** ** value**
Continuous									
BRI	12,231	1.22 (1.19–1.26)	< 0.001	1.21 (1.17–1.24)	< 0.001	1.21 (1.18–1.25)	< 0.001	1.11 (1.08–1.15)	< 0.001
Categories									
T1 (≤ 3.7)	3984	1 (ref)		1 (ref)		1 (ref)		1 (ref)	
T2 (> 3.7 and ≤ 4.7)	4143	1.4 (1.28–1.54)	< 0.001	1.36 (1.24–1.49)	< 0.001	1.4 (1.27–1.53)	< 0.001	1.25 (1.13–1.37)	< 0.001
T3 (> 4.7)	4104	1.86 (1.7–2.04)	< 0.001	1.78 (1.62–1.95)	< 0.001	1.82 (1.65–1.99)	< 0.001	1.42 (1.28–1.57)	< 0.001
Trend test			< 0.001		< 0.001		< 0.001		< 0.001

*Note:* Model 1: sex and age. Model 2: sex, age, education, duration of diabetes, family history of diabetes, smoking, and alcohol consumption. Model 3: sex, age, education, duration of diabetes, family history of diabetes, smoking, alcohol consumption, HOMA-IR, HbA1c, hypertension history, and dyslipidemia history.

Abbreviations: BRI, body roundness index; CI, confidence interval; DKD, diabetic kidney disease; OR, odds ratio; Ref, reference; T, tertile.

## Data Availability

The datasets of the study are available from the corresponding author upon reasonable request.
