# Supplementary material for: Relationship Between Body Roundness Index and Diabetic Kidney Disease in Patients With Type 2 Diabetes Mellitus: A Population-Based Study
Source: J Diabetes Res. 2025 Sep 3;2025:1854458. doi: 10.1155/jdr/1854458 (PMC12422861; doi:10.1155/jdr/1854458)
Supplement: Supporting Information 1 — Additional supporting information can be found online in the Supporting Information section. Table S1: Demographic and clinical parameters of study patients. [file 1854458.f1.docx]

Table S1. Demographic and clinical parameters of study patients

| Variables | BRI | | | | |
| --- | --- | --- | --- | --- | --- |
|  | Total | T1 (≤3.7) | T2 (>3.7, ≤4.7) | T3 (>4.7) | p-Value |
| No. | 12231 | 3984 | 4143 | 4104 |  |
| Sex, n (%) |  |  |  |  | < 0.001 |
| Female | 4835 (39.5) | 1304 (32.7) | 1535 (37.1) | 1996 (48.6) |  |
| Male | 7396 (60.5) | 2680 (67.3) | 2608 (62.9) | 2108 (51.4) |  |
| Age (y) | 55.0 (47.0, 63.0) | 53.0 (46.0, 60.0) | 55.0 (48.0, 63.0) | 58.0 (48.0, 66.0) | < 0.001 |
| Education, n (%) |  |  |  |  | < 0.001 |
| Below high school | 10193 (83.4) | 3189 (80) | 3473 (83.8) | 3531 (86.1) |  |
| High school education and above | 2035 (16.6) | 795 (20) | 670 (16.2) | 570 (13.9) |  |
| DBP (mmHg) | 75.0 (69.0, 83.0) | 74.0 (68.0, 81.0) | 76.0 (69.0, 84.0) | 77.0 (70.0, 84.2) | < 0.001 |
| SBP (mmHg) | 130.0 (120.0, 141.0) | 125.0 (116.0, 136.0) | 130.0 (121.0, 141.0) | 134.0 (125.0, 146.0) | < 0.001 |
| BMI (kg/m2) | 25.2 (23.1, 27.6) | 22.5 (21.0, 24.0) | 25.3 (23.9, 26.6) | 28.3 (26.4, 30.5) | < 0.001 |
| WC(cm) | 89.2 (83.0, 96.0) | 81.0 (76.8, 85.0) | 89.3 (86.0, 93.0) | 98.0 (94.0, 103.0) | < 0.001 |
| HC(cm) | 95.0 (90.4, 100.0) | 90.0 (87.0, 94.0) | 95.0 (92.0, 98.1) | 100.0 (96.0, 105.0) | < 0.001 |
| WHR | 0.9 (0.9, 1.0) | 0.9 (0.9, 0.9) | 0.9 (0.9, 1.0) | 1.0 (1.0, 1.0) | < 0.001 |
| BRI | 4.2 (3.5, 5.1) | 3.2 (2.8, 3.5) | 4.2 (4.0, 4.5) | 5.5 (5.1, 6.1) | < 0.001 |
| VFA(cm2) | 94.0 (65.9, 123.7) | 62.0 (39.0, 85.0) | 95.0 (74.0, 116.0) | 126.0 (102.0, 154.0) | < 0.001 |
| Duration of diabetes (y) | 3.4 (0.2, 10.2) | 3.6 (0.2, 10.1) | 3.2 (0.2, 10.0) | 3.4 (0.2, 10.3) | 0.249 |
| History of hypertension, n (%) | 5517 (45.1) | 1184 (29.7) | 1911 (46.1) | 2422 (59) | < 0.001 |
| History of dyslipidemia, n (%) | 5517 (45.1) | 1184 (29.7) | 1911 (46.1) | 2422 (59) | < 0.001 |
| Family history of diabetes, n (%) | 5840 (47.7) | 2027 (50.9) | 2022 (48.8) | 1791 (43.6) | < 0.001 |
| Smoking，n (%) | 3591 (29.5) | 1336 (33.7) | 1238 (30.1) | 1017 (24.9) | < 0.001 |
| Drinking，n (%) | 4284 (35.2) | 1528 (38.5) | 1515 (36.8) | 1241 (30.4) | < 0.001 |
| FBG (mmol/L) | 8.2 (6.6, 11.1) | 8.2 (6.6, 11.6) | 8.2 (6.7, 10.9) | 8.2 (6.6, 10.8) | 0.147 |
| FCp(ng/mL) | 2.1 (1.5, 2.9) | 1.7 (1.2, 2.3) | 2.1 (1.6, 2.8) | 2.6 (1.9, 3.4) | < 0.001 |
| HOMA-IR | 3.6 (2.9, 4.7) | 3.2 (2.6, 4.1) | 3.7 (3.0, 4.7) | 4.1 (3.3, 5.3) | < 0.001 |
| HbA1c (%) | 8.2 (6.9, 10.2) | 8.5 (6.9, 11.0) | 8.1 (6.9, 10.1) | 8.1 (6.9, 9.8) | < 0.001 |
| UN (mmol/L) | 5.3 (4.4, 6.5) | 5.3 (4.4, 6.4) | 5.3 (4.4, 6.5) | 5.3 (4.3, 6.5) | 0.815 |
| Scr (mmol/L) | 63.0 (52.0, 76.0) | 63.0 (53.0, 75.0) | 64.0 (53.0, 76.0) | 62.0 (51.0, 76.0) | 0.002 |
| e-GFR (mL/min per 1.73 m2) | 104.4 (85.4, 125.5) | 106.8 (89.1, 127.4) | 103.5 (85.2, 124.2) | 103.1 (82.1, 125.1) | < 0.001 |
| UA (mmol/L) | 328.0 (268.0, 397.0) | 309.0 (254.0, 370.0) | 333.0 (273.0, 399.0) | 342.0 (282.0, 418.0) | < 0.001 |
| TG (mmol/L) | 1.6 (1.1, 2.4) | 1.3 (0.9, 2.0) | 1.6 (1.1, 2.5) | 1.7 (1.2, 2.5) | < 0.001 |
| TC (mmol/L) | 5.1 (4.3, 5.9) | 5.0 (4.2, 5.8) | 5.1 (4.3, 5.9) | 5.1 (4.3, 6.0) | 0.003 |
| HDL-C (mmol/L) | 1.1 (0.9, 1.3) | 1.1 (1.0, 1.4) | 1.1 (0.9, 1.3) | 1.1 (0.9, 1.3) | < 0.001 |
| LDL-C (mmol/L) | 2.9 (2.2, 3.5) | 2.9 (2.2, 3.5) | 2.9 (2.3, 3.5) | 2.9 (2.2, 3.6) | 0.475 |
| UACR（mg/g） | 20.1 (8.9, 58.7) | 16.0 (7.5, 43.3) | 19.5 (8.9, 58.1) | 26.0 (11.0, 75.4) | < 0.001 |
| DKD, n (%) | 5020 (41.0) | 1329 (33.4) | 1710 (41.3) | 1981 (48.3) | < 0.001 |

Note: Data are presented as counts(%) or medians (interquartile ranges). Non-normally distributed continuous data were analyzed using the Mann–Whitney U test, and the Chi-squared test was used for comparing categorical data.

Abbreviations: DKD, diabetic kidney disease; SBP, systolic blood pressure; DBP, diastolic blood pressure; BMI, body mass index; WC, waist circumference; WHR, waist-to-hip ratio, VFA, visceral fat area, FBG, fasting blood glucose; FCp, fasting serum C peptide; HOMA-IR, homeostasis model assessment of insulin resistance; HbA1c, glycated hemoglobin; UN, urea nitrogen; Scr, serum creatinine; eGFR, estimated glomerular filtration rate; UA, uric acid; TG, triglycerides; TC, total cholesterol; HDL-C, high-density lipoprotein cholesterol; LDL-C, low-density lipoprotein cholesterol; UACR, urinary albumin-to-creatinine ratio.
